# Sun Protection Used by Cyclists in Southern Brazil

**DOI:** 10.1111/jocd.70049

**Published:** 2025-02-13

**Authors:** Eduarda Mazurkievz de Freitas, Kátia Sheylla Malta Purim

**Affiliations:** ^1^ Universidade Positivo (UP) Curitiba Brazil

**Keywords:** environmental exposure, photoaging, photoprotection, skin cancer, sunburn, sunscreens

## Abstract

**Background:**

Cycling is a practice that is increasing globally. However, there is a paucity of studies regarding sun protection usage among cyclists.

**Methods:**

The present study uses a cross‐sectional design. An online survey was conducted using the Google Forms platform, and a self‐administered form was used to gather the participant's sociodemographic details, sporting practices, and sun protection usage habits. The full questionnaire file can be found as Supporting Information. Descriptive statistics were analyzed using Microsoft Excel, and the chi‐square test of independence was performed to determine the association between the variables using a contingency table.

**Results:**

The study sample comprised 379 cyclists, predominantly men (69.7%), and most cyclists were observed to be using some type of cycling‐related application (81.3%). Approximately 67.3% of the cyclists used sunscreen. The sunscreen was applied once a day (45.4%), and the usage of sunscreen was lower in men (58%) and those with higher phototypes (69.2%). Furthermore, using long‐sleeved shirts and sunglasses while cycling was 49.3% and 75.5% of the participants, respectively. Among the participants who did not use sun protection, 35% cycled for over 7 h a week.

**Conclusion:**

The results indicate that cyclists must be encouraged to use sun protection, especially men and athletes with higher phototypes. Such use could be optimized in this population, associating the technologies related to the practice of cycling with health promotion.

## 1 Introduction

Cycling is an easily accessible sport that has various physical and mental health benefits. Evidence from large prospective cohort studies indicates that regular cycling is associated with a lower risk of mortality [1, 2]. Moreover, it can be recommended as a factor that promotes longevity and improved quality of life, inclusion, socialization, overall well‐being, and sustainable mobility.

As with any other outdoor activity, cycling is associated with exposure to the sun, and therefore, to ultraviolet (UV) radiation. A prospective cohort study conducted in Sweden by Lindqvist et al. at the Karolinska Institute, which began in 1990 and involved approximately 30 000 women, reported that sun exposure was the most prominent external factor for the development of skin cancer, melanoma, and basal cell and squamous cell carcinomas [3].

Globally, over three million cases of skin cancer are diagnosed annually, and individuals who work outdoors or who practice an outdoor hobby or sport involving regular sun exposure are among those at a higher risk for the disease. The global incidence of skin carcinoma caused by occupational sun exposure is expected to increase by 60%, with no significant differences being observed in regard to gender or geographic location [4].

The Helios Project, after studying individuals who were regularly exposed to the sun, reported a higher incidence of sunburn and a twofold higher frequency of melanoma, carcinoma, and precancerous lesions compared with a population that did not work outdoors, have a hobby, or play sports involving exposure to the sun. Moreover, 40% of the group with greater exposure to the sun did not use any sunscreen when outdoors [4].

Research has shown that sun exposure leads to the development of other skin conditions as a result of UV light exposure, including melasma and photoaging [5, 6]. It has also been shown that the duration of exposure is proportional to the severity of melasma, with many patients not being aware of this causality and relationship [5]. Sun protection is the main preventive and therapeutic strategy against sunburn, photoaging, and skin cancer.

A study conducted in 2022 on the use of sunscreen in Brazil showed that 71% of Brazilians do not use sun protection on a daily basis, and 55% are not aware of how much sun protection should be applied. Furthermore, 57% of people who use sunscreen do not reapply it during the day, and only 12% wear clothes that can act as a protective barrier [7]. These findings are alarming and confirm those of a study conducted in the USA, which showed that only 49.5% of the sample population used sunscreen when exposed to high levels of radiation, and 66.5% believed that information about the use of sunscreen use should be provided in schools [8].

Wearable technologies and applications (apps) are increasingly used by athletes and nonathletes, as aids for raising awareness regarding sun protection usage habits [8, 9]. However, little is known about sun protection among cyclists in Brazil. Therefore, this study analyzes this topic and compares levels of sun exposure and sun protection usage habits between men and women.

## 2 Methods

This was an analytical cross‐sectional study, with cycling athletes as the target study population. The study was approved by the human research ethics committee of Universidade Positivo (CAAE).

The calculated sample size was 377 individuals, for a sample power higher than 95%. Participants were both men and women over 18 years of age, and there was no restriction on color/race/ethnicity or cycling category of the participants. Furthermore, an informed consent form was signed by all participants.

Potential participants were recruited from cycling groups present on Facebook, WhatsApp, and Instagram. The participants filled out an online Google Forms questionnaire prepared by the researchers, which included questions related to sociodemographic aspects (sex, age, level of education, color/race/ethnicity), cycling practice (e.g., motivation, duration and frequency, participation in competitions), sun exposure and protection, (skin sensitivity, personal and/or family history of skin cancer, use of sunscreen, wearing a long‐sleeved shirt, and wearing sunglasses), and episodes of sunburn.

Skin color (White, Mixed Race, Asian, and Black) was self‐reported and skin sensitivity and reaction to sun exposure was determined according to the Fitzpatrick classification. The question regarding sunscreen usage asked about the sun protection factor (SPF), how many times it was applied each day, and the areas of the body to which it was applied. The sun protection measures considered included wearing of sports garments and accessories such as sunglasses.

Data were analyzed using Microsoft Excel software, 2021 version. The chi‐square test of independence was used to investigate any association between the variable in the row and the variable in the column of a contingency table constructed from the sample data; significance level was set at *p*‐value of < 0.05.

## 3 Results

Overall, 379 cyclists aged 18–76 years (SD = 13.42) participated in the study; 264 were men (69.7%) and 115 were women (30.3%). Majority of the participants had completed either secondary (36.3%) or higher education (22.9%; Table 1).

**TABLE 1:** Sociodemographic characteristics of the cyclists (*n* = 379).

	Total *n* = 379 (100% of the sample)
Gender	
Female	115 (30.3%)
Male	264 (69.7%)
Color/race/ethnicity	
White	249 (65.7%)
Mixed race	60 (5.9%)
Black	26 (6.9%)
Asian	6 (1.5%)
Level of education	
Secondary education	81 (36.3%)
Higher education	51 (22.9%)
Postgraduation	42 (18.8%)
Incomplete higher education	28 (12.6%)
Incomplete secondary education	16 (7.2%)
Participates in official cycling competitions	
Yes	145 (38.3%)
No	234 (61.7%)
Recreational/hobby	124 (32.7%)
Transport and hobby	65 (17.2%)
Nonsponsored sport	21 (5.5%)
Ecotourism	20 (5.3%)
1 time	37 (9.8%)
2 times	70 (18.5%)
3 times	112 (29.6%)
4 times	63 (16.6%)
5 times	31 (8.2%)
6 times	28 (7.4%)
7 times	26 (6.9%)
Over 10 h	52 (13.7%)

Majority of the participants were White (65.7%). A total of 115 participants (30.3%) had high skin sensitivity and burned easily when exposed to the sun; 28 (7.4%) reported a history of skin cancer.

Cycling was practiced as a hobby by 32.7% and as a nonsponsored sport by 35.9% of the participants. The weekly frequency of cycling was twice a week (18.5%) and three times a week (29.6%).

Considering the total hours reported to be spent cycling, 190 of the 379 (50.1%) cyclists reported that they spent less than 5‐h cycling, whereas 189 (49.9%) reported that they spent more than 5‐h cycling. Furthermore, 145 athletes (38.3%) participated in competitions.

With regard to sun protection measures, 255 (67.3%) of the cyclists reported using sunscreen, 286 (75.5%) wore sunglasses, and 187 (49.3%) wore a long‐sleeved shirt while cycling.

Among the 255 cyclists who used sunscreen, this use was higher among Asian (83.3%) and White cyclists (73.5%), accounting for 183 of the 255 respondents. The association between skin type and use of sunscreen was accepted for a *p*‐value of < 0.05 for a level of confidence of 95% and 3 degrees of freedom.

Women were more likely to use sunscreen (88.7%), whereas most of the men did not use any (89.5%). With a *p*‐value of < 0.05 for a level of confidence of 95% and 1 degree of freedom, an association between the use of sunscreen and gender is accepted.

The body areas to which the cyclists applied sunscreen were the face (75.2%), ears (43.4%), and arms (31.1%). SPF 30 or higher was used, and there was a preference for industrial products (75.7%), which have been produced and processed by large cosmetics industries. Sunscreen was predominantly applied once a day (45.4%; Table 2).

**TABLE 2:** Characteristics of sunscreen usage by cyclists.

	Total *n* = 255 (67.3% of the sample)
Gender	
Female	102 (88.7%)
Male	153 (58%)
Color/Race/Ethnicity	
White	183 (73.5%)
Mixed race	49 (50%)
Black	18 (69.2%)
Asian	5 (83.3%)
Frequency of application during the day	
1 time	172 (45.4%)
2 times	66 (17.4%)
3 times	17 (4.5%)
Sun protection factor (SPF)	
SPF 30 or higher	287 (75.7%)
SPF lower than 30	42 (11.1%)
When exposed to the sun, your skin	
Burns easily and never tans	43 (11.3%)
Burns easily and tans poorly	72 (19%)
Burns and tans moderately	121 (31.9%)
Burns minimally and tans easily	96 (25.3%)
Never burns and tans intensely	47 (12.4%)
Preferred origin of cosmetic products	
No preference	181 (47.8%)
Industrial	128 (33.8%)
Compounded	16 (4.2%)
Natural	44 (11.6%)
Vegan	9 (2.4%)
Hypoallergenic	18 (4.7%)
Areas to which sunscreen applied	
Face	255 (67.2%)
Ears	164 (43.3%)
Lips	77 (20.3%)
Nape	120 (31.7%)
Chest	42 (1.8%)
Abdomen	18 (4.7%)
Arms	118 (31.1%)
Backs of the hands	101 (26.6%)
Legs	96 (25.3%)
Ever had sunburn	
Yes	65 (17%)
No	314 (83%)
Personal history of skin cancer or premalignant lesion	
Yes	28 (7.4%)
No	351 (92.6%)

An important finding was that the athletes who did not wear sunscreen accounted for a third of those who cycled more than 7 h a week, and therefore, were more susceptible to damage from UV radiation (frequent practice leading to greater exposure).

Notably, majority of those who applied sunscreen more than three times a day cycled the highest number of hours—over 5 h (65%), and few of the respondents (4.5%) used sunscreen extremely frequently (three or more times a day).

Prevalence of at least one episode of sunburn caused by sun exposure when cycling was 17% in 65 respondents. Furthermore, sunburn occurred more frequently in White and Asian cyclists (21%) and less frequently in Mixed Race and Black cyclists (9%). An association between skin type and sunburn was accepted for a *p*‐value of < 0.05 for a level of confidence of 95% and 3 degrees of freedom.

As demonstrated in Table 3, among those who had experienced sunburn (17%), the percentage of participants who used sunscreen (18%) was similar to that of those who did not (15.3%). An association between sunburn and the use of sunscreen was not found for a *p*‐value of > 0.05 for a level of confidence of 95% and 1 degree of freedom.

**TABLE 3:** Frequency of sunburn in cyclists according to color/race/ethnicity, use of sunscreen, and frequency of application during the day.

	Total *n* = 65 (17% of the total sample)
Color/race/ethnicity	
White	53 (81.5%)
Mixed Race	10 (15.4%)
Black	1 (1.5%)
Asian	1 (1.5%)
How many times is sunscreen applied during the day	
1 time	33 (71.7%)
2 times	12 (26.1%)
3 times	1 (2.2%)
Use of sunscreen	
Yes	46 (70.8%)
No	19 (29.2%)

However, among those who had experienced sunburn, the number of cases was higher among men and women who used sunscreen only once a day (19.2%). Incidences of sunburn decreased with increasing frequency of sunscreen use, occurring at a rate of only 5.9% in the group that used sunscreen three times a day. With *p*‐value of > 0.05 for a level of confidence of 95% and 3 degrees of freedom, an association between the frequency of sunscreen use and sunburn is not accepted.

Sunglasses were worn by 75.5% of the cyclists; long‐sleeved shirts were worn by 49.3%, sunscreen (once a day) was used by 45.4%, and sunscreen with SPF 30 or higher was used by 75.7%. Furthermore, 51.5% of participants cycled before 10 am, and 22.7% consulted the Weather app. Strava was the most commonly used free app for planning cycling activities (81.3%).

## 4 Discussion

Analysis of sunscreen use among the participants showed a lower adherence among men and Black cyclists, which is in line with the literature [10, 11, 12] and indicates that men do not use skincare products as much as women. A study involving 705 men reported that 83% of them did not use sunscreen every day, and that men who self‐identified as Black or African–American were less likely to use it [10].

These data are alarming, considering that men are more prone to immunosuppression induced by UV radiation; moreover, men's skin does not respond as well women's skin to environmental stress factors like UV rays, and they are twice as more likely to develop skin cancer during their lifetime [10]. The American Cancer Society predicts that in 2024, men will account for 59.3% of new cases of skin cancer (excluding nonmelanoma cancer) and 66.3% of deaths caused by these tumors. Most cases of melanoma can be prevented by reducing sun exposure, taking measures such as using broad‐spectrum sunscreen with SPF 30 or higher, wearing protective clothing, staying in the shade, and avoiding sun exposure at midday [11].

Research suggests that men are more likely to use sunscreen when warned regarding the risks of skin cancer, and when the products are adapted to their skin type [10]. The use of sunscreen is higher among women, as was the case in the present study, and the study conducted in New Orleans showed a difference in sunscreen usage between men and women of approximately 20% [13].

The lower adherence observed among Black individuals is also in line with the literature [12]. The factor that appears to influence the use of sunscreen the most in this group is its cosmetic role; however, majority of dermatologists do not talk about sunscreen usage in patients with darker skin, and this might have led to lower use of sunscreen among these individuals [12]. However, sunscreen usage is also essential for the complete care of patients with higher phototypes, between V (medium to dark brown skin color) and VI (deep brown to black skin color) according to the Fitzpatrick Scale, because, despite its different features, the skin can potentially develop tumors or other skin conditions that worsen with sun exposure.

In the present study, with regard to the use of sunscreen, frequency of application, and the occurrence of sunburn, 45.4% of the cyclists applied sunscreen only once a day, which does not offer total protection. According to the American Academy of Dermatology, to ensure effective protection, sunscreen should be reapplied every 2 h when outdoors, as it is lost through sweating. It should also be reapplied after swimming [14].

As cycling is an activity that results in significant sweating and increased skin friction via physical contact, sunscreen should be immediately reapplied to compensate for the loss of protective effect. For adequate coverage, 2 mg/cm^2^ of sunscreen should be applied. However, studies show that consumers usually apply much less than is recommended (between 0.5 and 1.5 mg/cm^2^). The main factor for the efficacy of sunscreen is applying the right amount [15]. Incorrect and insufficient sunscreen application can lead to a false sense of sun protection; prolonged sun exposure; and increased risk of burns and skin spots, aging, and cancer [16].

One of the main points analyzed in the present study was the relationship between hours spent cycling and sunscreen use. About one third of the cyclists who cycled for more than 7 h per week did not apply any sunscreen. Thus, it is imperative to raise awareness regarding the effects of UV radiation from sun exposure, as there are few studies that warn of this danger. Recent studies indicate that the incidence of basal cell and squamous cell carcinoma is higher among fair‐skinned workers exposed to sunlight. Athletes who spend more time in the sun, for example, marathon runners, have a higher risk of developing melanoma [17].

Research has shown that several outdoor sports are risk factors for skin cancer. This type of activity is associated with increased sun exposure and the occurrence of sunburn, which can lead to melanoma and nonmelanoma cancer. High‐intensity training can cause lesions in the tissues and induce immunosuppression. Additionally, sweating when engaged in sports can increase photosensitivity and exacerbate the harmful effects of UV radiation [18].

Loria et al. analyzed the risk factors for the development of melanoma among athletes, including cyclists, and showed that those who were exposed more to the sun as a result of sports activity were more likely to develop melanoma [19].

When athletes do not use sunscreen, their skin ages, as chronic sun exposure leads to changes in epidermal thickness, increased heterogeneity of pigmentation and dermal elastosis, degradation of collagen in the dermis, development of vascular ectasias, and increased mutagenesis of keratinocytes and melanocytes in the skin [20].

In a prospective study where participants used sunscreen SPF 30 every day for 52 weeks, clinical evaluations showed that all parameters of photoaging had improved significantly, from the beginning of week 12 until week 52. Skin texture, clarity, and mottled and discrete pigmentation were the parameters that exhibited the most improved by the end of the study (40%–52% improvement from baseline), with 100% of individuals showing improved skin clarity and texture [21].

Outdoor physical activity exposes athletes to the cumulative action of sunlight, especially younger athletes, who should maximize sun safety to avoid short‐term and long‐term skin damage. Sun protection should be used throughout the lifetime to mitigate the negative effects of radiation, even on cloudy days [19].

A study on the perceptions of 927 recreational cyclists about the risks of skin cancer and sun protection measures reports that when they were made aware, they realized the advantages of sunscreen application and perceive it as easy and viable, intending to use it more often [22]. This demonstrates the importance of disseminating information and promoting measures of protection and prevention that are more centered on the benefits for the individuals and their needs.

The use of physical barriers for sun protection, such as protective clothing, reduces the development of nevi. However, not all materials offer sufficient sun protection. Moreover, the protection factor depends on the type, density, color, and design of the material, and the processes used in finishing it. The use of darker dyes in thick materials provides more adequate protection [23].

In the present study, 49.3% and 75.5% of individuals wore long‐sleeved shirts and sunglasses, respectively. In a study with 830 individuals, the percentage of individuals who wore sunglasses and protective clothing was 50%, showing good adherence to these practices and their viability [24].

The limitations of the present study were its cross‐sectional design and the potential bias of information, because the data were self‐reported by the respondents. Nevertheless, this study contributes to the health of cyclists, raises awareness about the need for these athletes to improve their sun protection practices, and indicates that these practices can be improved with the use of technology.

Due to the amount of sun exposure experienced by cyclists, it is important to encourage institutions that organize cycling events and cycling educators to promote educational campaigns about sun exposure and skin cancer. An important finding of this study was the frequent use of apps to record cycling data. These could also be used as platforms to provide educational messages about skin care, focusing primarily on the male population, particularly men with higher phototypes. These apps can also be used to promote correct sunscreen application methods, providing information about the amount of product and number of reapplications requires, and to provide details about available sun protection resources such as physical barriers, namely long‐sleeved shirts, and sunglasses.

## 5 Conclusion

In the group of cyclists analyzed in this study, sun protection was shown to be inadequate, especially among men and Black cyclists. Thus, the available sun protection measures should be more disseminated and promoted, and behaviors that can protect against sun exposure should be incorporated into apps that are routinely used by cyclists, such as those that record cycling data.

## Author Contributions

**Eduarda Mazurkievz de Freitas:** conceived and designed the analysis; data collection; contributed data/analysis tools; performed the analysis; wrote the paper. **Kátia Sheylla Malta Purim:** conceived and designed the analysis; data collection; supervision of the work.

## Ethics Statement

Human subjects: Consent was obtained or waived by all participants in this study. Comitê de Ética em Pesquisa em Seres Humanos da Universidade Positivo (CAAE) issued approval 58292022.8.0000.0093. Animal subjects: All authors have confirmed that this study did not involve animal subjects or tissue.

## Conflicts of Interest

All authors declare the following: Payment/services info: All authors have declared that no financial support was received from any organization for the submitted work. Financial relationships: All authors have declared that they have no financial relationships at present or within the previous 3 years with any organizations that might have an interest in the submitted work. Other relationships: All authors have declared that there are no other relationships or activities that could appear to have influenced the submitted Word.

## Supporting information


Data S1.

## Data Availability

All of the data supporting underlying findings are included in the manuscript and its Data S1.
