# Supplementary material for: Sun Protection Used by Cyclists in Southern Brazil
Source: J Cosmet Dermatol. 2025 Feb 13;24(2):e70049. doi: 10.1111/jocd.70049 (PMC11822412; doi:10.1111/jocd.70049)

RESEARCH TITLE: **"ANALYSING THE SKIN CARE OF CYCLISTS"
DATA COLLECTION INSTRUMENT -** applied after consent by the participant.

**TERM OF FREE AND INFORMED CONSENT (TCLE)
RESEARCH TITLE: "ANALYSING THE SKIN CARE OF CYCLISTS"**

**DIGITAL INFORMED CONSENT FORM :**

**You are being invited via social media as a volunteer to take part in the research "ANALYSIS OF CUTANEOUS CARE OF CYCLISTS". The aim of the research is to assess the skin self-care of cyclists with diabetes, compared with healthy cyclists matched on gender and age. It should be noted that the data collected will be kept confidential and in the event of publication, no participant's identity will be revealed, and respect and anonymity will be ensured for their responses. Participants will not be subjected to any physical risks. There will be no interventions, no conflicts of interest or funding and no data will be collected that identifies the participants. As this is a digital survey, there may be a risk of losing the secrecy and confidentiality of the data, but in order to prevent this, the researcher guarantees that only she will have access to the data collected, the data will only be handled on one computer, and then destroyed after the project has ended, and a data secrecy and confidentiality agreement, which states that all care and proper treatment of the data will be taken, signed by the researcher, has been attached to the project. It is also worth emphasising that there will be no direct benefit to the research participant. The results could be useful for improving knowledge about the self-care of diabetic athletes, planning and developing educational actions to improve health training and encourage the prevention of skin lesions. Your participation in this study is voluntary and if you no longer wish to take part in the research you can withdraw at any time and ask to be returned the signed informed consent form. If you have any questions, you can also contact the Research Ethics Committee (CEP) of Universidade Positivo - Rua Professor Pedro Viriato Parigot de Souza 5300, campus headquarters - Ecoville, 2nd floor of the Central Library, Curitiba-PR, telephone (41) 3317-3260 e-mail: <cep@up.edu.br> or the National Research Ethics Committee (CONEP) Esplanada dos Ministérios, bloco G - edifício Anexo - Ala "B" - 1o andar - sala 103 B - CEP 70058-900 Brasília - DF, telefone (61) 3315-2150/3315-3821 - e-mail cns@saude.gov.br. You are not responsible for the expenses necessary to carry out the research. Nor will there be any payment or gratuity for your participation in the research. I understand that I am free to discontinue my participation at any time without justifying my decision. If you do not wish to take part, please tick "NO" and if you agree to take part in the research, please click "YES" below. This digital form will be in two identical copies, and when you agree to take part, you will receive one copy of the form by e-mail, after which you will have access to the questionnaire on the following pages. Thank you in advance!**

**"I voluntarily agree to participate in this study and, in doing so, I understand that my identity will not be revealed and that my confidentiality will be maintained."**

*** Yes * No
Email _____________________________SIgnature._________________________________Date____/____/____**

**Obs - This project was approved by research ethics committee on 04/07/22 (opinion 5.506.827)**

**RESEARCH: " ANALYSING THE SKIN CARE OF CYCLISTS"
Questionnaire
Age____________ Gender**:🞏Female🞏Male🞏Other________________ **Weight:** _________ **Height**:____________ **Profession/Occupation__________________________________________ Colour/Race/Ethnicity**🞏White🞏Black🞏Brown🞏Yellow

**Level of education**

🞏Elementary school🞏Incomplete secondary school🞏Secondary school🞏Incomplete higher education🞏Complete higher education🞏Postgraduate studies

**City/State where you live____________________________________**

**You use cycling for:**

🞏Recreational / leisure🞏Transport🞏Transport and leisure🞏Sport without sponsorship🞏Sport with sponsorship🞏Ecotourism **How long have you been cycling?**

🞏< 1 year🞏1 to 3 years🞏4 to 6 years🞏7 to 9 years🞏10 to 15 years🞏16 to 20 years🞏> 20 years **How many times do you cycle a week?**

🞏1 time🞏2 times🞏3 times🞏4 times🞏5 times🞏6 times🞏7 times🞏Currently none

**How many kilometres do you cycle a week?**

🞏1 to 10km🞏11 to 20km🞏21 to 30km🞏31 to 40km🞏41 to 50km🞏51 to 60km🞏61 to 70km🞏71 to 80km🞏81 to 90km 🞏91 to 100km🞏101 to 120km🞏121 to 150km🞏151 to 170km🞏171 to 200km🞏> 200km🞏currently not cycling

**How many hours do you cycle a week?**

🞏< 1h🞏1 to 2h🞏2 to 3h🞏3 to 4h🞏4 to 5h🞏5 to 6h🞏7 to 8h🞏9 to 10h🞏> 10 hours
**Do you cycle more than once a day?**🞏Yes🞏No
**What is your average training speed (intensity)?**🞏< 10Km🞏10 to 15 km🞏15 to 20 km🞏20 to 25 km🞏> 25 km **Do you take part in official cycling competitions?**🞏Yes🞏No
**Have you ever had a cycling injury?**🞏Yes🞏No

**If you answered yes about limiting cycling injuries, which injuries:**

🞏back pain🞏heel pain🞏fractures🞏tendonitis🞏knee pain🞏hip pain🞏ankle pain🞏foot pain🞏muscle sprain/strain🞏 Other_____________

**Do you have a history of orthopaedic or trauma surgery related to cycling?**🞏Yes🞏No
**Have you ever had - Sunburn (face, ear, nose, lip, shoulder, other)?**🞏Yes🞏No
**Have you ever had - Rashes (armpits, groin, other)?** 🞏Yes🞏No
**Have you ever had - Blisters (heel, plantar region, back of toes, other)?** 🞏Yes🞏No
**Have you ever had - Calluses (back of toes, plantar region, palmar region, other)?**🞏Yes🞏No **Have you ever had abrasions or cuts (upper limbs, lower limbs, face, other)?**🞏Yes🞏No **Have you ever had - Haematomas (upper limbs, lower limbs, face, other)?**🞏Yes🞏No

**Have you ever had - Insect bites (upper limbs, lower limbs, face, other)?**🞏Yes🞏No

**Have you ever had other types of skin or mucous membrane problems related to sport? If yes, please specify**

**_________________________________________________________________________________**

**Times most used for training:**🞏before 10 a.m.🞏between 10 a.m. and 4 p.m.🞏after 4 p.m.

**Do you often wear sunglasses?**🞏Yes🞏No

**Do you often wear long-sleeved shirts?**🞏Yes🞏No

**In relation to the sun, your skin:**🞏"burns easily and never tans"🞏"burns easily and tans discreetly" 🞏"burns and tans moderately"🞏"burns little and tans a lot"🞏"never burns and tans intensely**"**

**Frequency of sunscreen use:**🞏1 time/day🞏2 times/day🞏3 times/day or more🞏No use

**Region of the body where you apply sunscreen:**🞏face🞏ears🞏nose🞏lips🞏nape🞏torso🞏abdomen🞏upper limb🞏back of hands🞏lower limbs🞏no region

**Sun protection factor used:**🞏SPF below 30🞏SPF 30 or more

**Do you have a personal history of skin cancer or premalignant lesions?**🞏Yes🞏No

**What is your preferred source of cosmetic products?**

🞏Industrialised🞏Manipulated🞏Natural🞏Vegan🞏Hypoallergenic🞏I have no preference

**Which free app(s) do you usually consult to plan your cycling activities?**🞏Climatempo,🞏UV Index 🞏Strava🞏Endomodo🞏Sportstracker🞏Moutains Bike Runstastic🞏Cycledroid🞏Cyclingnews🞏Bikermap🞏Other...


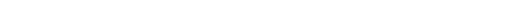

Supplement: Supplementary file 1 — Data S1. [file JOCD-24-e70049-s001.docx]
